# Analysis of Monkeypox Virus Exposures and Lesions by Anatomic Site

**DOI:** 10.3201/eid3011.241120

**Published:** 2024-11

**Authors:** Sarah Anne J. Guagliardo, Teresa Smith, Davidson H. Hamer, Ralph Huits, Phyllis Kozarsky, Michael Libman, Andrea M. McCollum, Kristina M. Angelo, GeoSentinel Network Collaborators

**Affiliations:** Centers for Disease Control and Prevention, Atlanta, Georgia, USA (S.A.J. Guagliardo, T. Smith, A.M. McCollum, K.M. Angelo); Boston University Chobanian & Avedisian School of Medicine, Boston, Massachusetts, USA (D.H. Hamer); Boston University Center on Emerging Infectious Diseases, Boston (D.H. Hamer); IRCCS Sacro Cuore Don Calabria Hospital, Verona, Italy (R. Huits); Emory University, Atlanta (P. Kozarsky); McGill University Health Centre, Montreal, Quebec, Canada (M. Libman)

**Keywords:** Mpox, monkeypox virus, exposure site, lesions, viruses, GeoSentinel Network, United States, Canada, Italy

## Abstract

We used cross-sectional data from 226 patients with monkeypox virus to investigate the association between anatomic exposure site and lesion development. Penile, anorectal, and oral exposures predicted lesion presence at correlating anatomic sites. Exposure site also predicted the first lesion site of the penis and anus.

Monkeypox virus (MPXV) can be transmitted from person-to-person through contact with mucous membranes, percutaneous exposures, or, less commonly, inhalation of infectious particles (*1*). In the 2022 global clade II mpox outbreak, most transmission was associated with sexual contact, particularly among men who have sex with men (*2*).

MPXV lesions begin as macules and papules and progress to vesicles and pustules (*3*). Lesions crust over and heal within 2–4 weeks (*4*). In recent outbreaks, a high prevalence and early appearance of anogenital lesions has been observed (*2*,*5*). A hypothesis that a rash will occur at the inoculation site has been proposed (*3*), yet the relationship between exposures and lesion site has not been studied in published literature to date.

GeoSentinel, a global epidemiologic surveillance network, collected data on 226 patients with MPXV in 2022. We used those data to evaluate the correlation between exposures and lesion presence, site of first appearance, and the number of lesions by anatomic site.

## The Study

We included in the study patients with a positive MPXV PCR test from skin or blood samples, who were >18 years of age and sought testing at a GeoSentinel site between May 1–July 1, 2022 (*5*). Although GeoSentinel surveillance typically includes only travel-associated cases, this study is from an enhanced surveillance project that included patients with and without international travel history. A questionnaire captured information about anatomic site of sexual exposures, physical examination, and underlying medical conditions. Healthcare practitioners completed the questionnaire by using medical record extraction and patient interviews. Lesion quantity was estimated ordinally on physical exam (1, 2–10, 10–50, 50–100, >100 lesions). Patients self-reported anatomic location of first lesions.

We focused our analysis on 3 anatomic sites (penis, anus, mouth) because there were sufficient patients exposed at these sites (>50%) to render statistical comparisons (*5*). We defined exposures at the rectum or anus as anorectal exposures and exposures at the oropharynx, including the mouth, oral mucosa, and pharynx, as oral exposures. MPXV lesion locations were described including at the penis, anus, oral mucosa, or lips. Rectal and oropharyngeal exams were not performed.

We calculated descriptive statistics for exposures and lesion outcomes. We evaluated the number of lesions by exposure site by using Mantel-Haenszel linear trend tests. For each anatomic site, we used univariable and multivariable logistic regression models to test the relationships between exposures, lesion presence, and site of lesion onset. We also adjusted multivariable models for other exposures and model assumptions. Participants recorded exposures by anatomic location as “yes” or “no/no response”; we assumed no response to indicate no exposure. To assess the influence of this assumption, we conducted a sensitivity analysis on 216 patients that reported sexual exposures and excluded patients who did not report sexual exposures.

Patients were from 15 countries, most of whom were from Spain (n = 79) and Canada (n = 66). All participants were assigned male sex at birth; median age was 37 (range 18–68) years, and 18% reported international travel in the 21 days before symptom onset (*5*). Most enrolled patients (99%) reported recent sexual contact with men, and 44% had HIV infection (median CD4 count 713 cells/mm^3^) (*5*). Those demographic characteristics are similar to those reported in large-scale surveillance analyses from Europe (*6*).

From 22% to 57% of patients had lesions at the exposure site; 10%–24% of patients reported that lesions first appeared at the exposure site (Figure). The number of penile lesions was significantly greater for patients exposed at the penis compared with patients not exposed at the penis (χ^2^ = 20.2; p<0.0001). The same pattern held for the anorectal exposures and anal lesions (χ^2^ = 22.7; p<0.0001), but we found no significant association for oral exposures and lesions.

**Figure:**
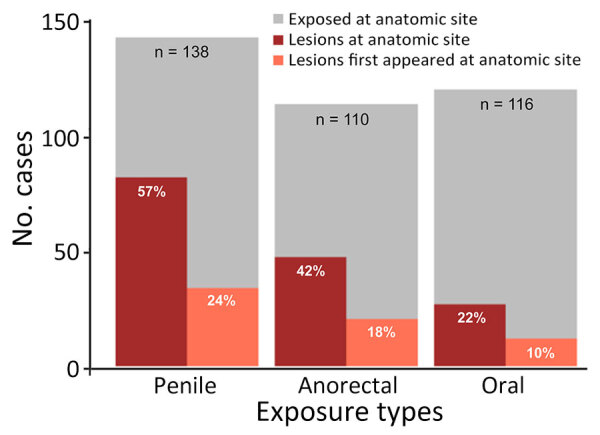
Locations of monkeypox virus exposures and locations and numbers of lesions reported by patients with monkeypox virus at GeoSentinel sites, May–July 2022. Percentages indicate numbers of patients who had lesions at the exposure site and those who reported the first lesion at the exposure site.

After accounting for other exposure types, the odds of having penile lesions were 14.6 (95% CI 5.6–45.0) times greater for patients with penile exposures compared with patients without penile exposures (Table 1). Anal lesions were 12.8 (95% CI 4.8–39.8) times more likely to occur among patients with anorectal exposures, and oral lesions were 5.4 (95% CI 1.7–19.7) times more likely to occur among patients with oral exposures.

**Table 1:** Characteristics of lesions and exposures among patients with monkeypox virus infection reported to GeoSentinel, May–July 2022*

Characteristics	Total no. responses	No. (%) responses		
		Yes†	No/not reported	Missing‡
Exposure site				
Penis	226	138 (61.1)	88 (38.9)	0
Mouth	226	116 (51.3)	110 (48.7)	0
Rectum/anus	226	110 (48.7)	116 (51.3)	0
Lesion site				
Penis	221	101 (45.7)	120 (54.3)	5 (2.2)
Mouth	218	60 (27.5)	158 (72.4)	8 (3.5)
Rectum/anus	221	43 (19.5)	178 (80.5)	5 (2.2)
Initial lesion onset site				
Penis	226	41 (18.1)	185 (81.9)	0
Mouth	226	30 (13.2)	196 (86.7)	0
Rectum/anus	226	25 (11.1)	201 (88.9)	0

*Exposures at each anatomic location were recorded as yes or no/no response. †Among complete responses. ‡Among all patients in dataset, N = 226.

The odds of developing penile lesions first were 7.3 (95% CI 2.7–21.2) times greater for persons with penile exposures after adjusting for other exposure types (Table 2). Among patients with anorectal exposures, lesions were 3.7 (95% CI 1.2–11.2) times more likely to first develop at the anus. There were no significant associations between oral exposures and first appearance of lesions. Sensitivity analyses revealed similar patterns (Appendix Table).

**Table 2:** Logistic regression models assessing the relationship between exposures, lesion presence or absence, and lesion onset site by anatomic site among patients with monkeypox virus who reported to GeoSentinel, May–July 2022*

No. pts.†	Outcome (no. pts.)‡	Predictor	No. pts.§	Univariable models					Multivariable models¶			
				OR	(95% CI)	p value	AIC		aOR	(95% CI)	p value	AIC
221	Penis lesions (101)	Penis exposure	138	3.7	(2.1–6.8)	<0.001	288.4		14.6	(5.6–45.0)	<0.0001	272.4
		Rectum/anus exposure	110	0.7	(0.4–1.2)	0.25	307.4		0.3	(0.1–0.7)	<0.01	
		Mouth exposure	116	1.2	(0.7–2.0)	0.59	308.5		0.4	(0.1–0.9)	0.056	
218	Anal lesions (60)	Rectum/anus exposure	110	4.8	(2.5–9.8)	<0.0001	236.84		12.8	(4.8–39.8)	<0.0001	229.4
		Penis exposure	138	0.9	(0.5–1.7)	0.76	260.4		0.3	(0.1–0.4)	<0.05	
		Mouth exposure	116	1.5	(0.8–2.7)	0.21	259		0.6	(0.2–1.9)	0.41	
221	Mouth lesions (43)	Mouth exposure	116	1.5	(0.8–3.0)	0.24	220.4		5.4	(1.7–19.7)	<0.01	216.2
		Penis exposure	138	0.7	(0.4–1.4)	0.31	220.8		0.3	(0.2–0.5)	<0.05	
		Rectum/anus exposure	110	0.9	(0.4–1.7)	0.63	221.6		0.5	(0.2–1.3)	0.17	
226	Penis lesions first (41)	Penis exposure	138	3.4	(1.4–7.6)	<0.01	209.4		7.3	(2.7–21.2)	<0.001	204.3
		Rectum/anus exposure	110	0.7	(0.3–1.4)	0.3	216.9		0.5	(0.2–1.1)	0.078	
		Mouth exposure	116	1.0	(0.5–2.0)	0.9	218.0		0.5	(0.2–1.3)	0.17	
226	Anus lesions first (30)	Rectum/anus exposure	110	2.4	(1.1–5.5)	0.038	176.4		3.7	(1.3–11.2)	<0.05	176.9
		Penis exposure	138	0.8	(0.4–1.8)	0.60	180.7		0.4	(0.1–1.2)	0.11	
		Mouth exposure	116	1.3	(0.6–2.8)	0.53	180.6		1.1	(0.3–3.9)	0.91	
226	Mouth lesions first (25)	Mouth exposure	116	0.9	(0.4–2.0)	0.72	161.1		3.1	(0.8–13.1)	0.11	158.9
		Penis exposure	138	0.5	(0.2–1.3)	0.16	159.4		0.4	(0.1–1.3)	0.15	
		Rectum/anus exposure	110	0.5	(0.2–1.1)	0.083	158.0		0.3	(0.1–1.0)	0.06	

*For each model, the main predictor variable of interest is listed first, followed by additional predictor variables. AIC, akaike information criterion; aOR, adjusted odds ratio; OR, odds ratio; pts., patients. †The number of observations included in the regression model. Missing observations: n = 5 for penis lesions, n = 8 for anus lesions, n = 5 for mouth lesions. ‡The number of patients who reported lesions at this anatomic site. §The number of patients who reported exposure at this anatomic site. ¶Covariates included in each multivariable model are all exposure types (e.g., for the outcome of penis lesions, penile, anorectal and oral exposures were included).

## Conclusions

Exposures to MPXV influence the clinical manifestations of disease (*7*), but little is known about the quantitative relationship between exposures and lesion development. Results from this analysis showed that penile, anorectal, and oral exposures were associated with lesion development and quantity at the same anatomic site. We found increased odds of lesions first appearing at the exposure site for the penis and anus but not for the mouth. This discrepancy between sites might be because practices that cause abrasions, such as condomless anal sex, might contribute to direct inoculation, increased viral exposure, and early development of mucosal lesions at the penis and anus or rectum.

Previous studies have reported that receptive anal sex was associated with anogenital lesions in men who have sex with men (*2*); vaginal and anal sex were associated with anogenital lesions in cisgender women and nonbinary persons (*8*). Similar phenomena have been observed previously with MPXV and other orthopoxviruses. MPXV-contaminated and vaccinia virus–contaminated needlestick exposures and animal bites and scratches from MPXV and cowpox-infected animals have caused initial lesions to develop at the site of inoculation (*7*,*9*–*11*).

Some patients did not have lesions reported at the exposure site. MPXV infection has a wide spectrum of clinical manifestations, ranging from mild to severe; some recent data suggest a small number of asymptomatic cases have occurred (*12*–*14*). It is possible lesions that were small, painless, and few might have been missed. In addition, our data only captured binary exposures by anatomic location but did not assess duration or nature of exposures, which might influence lesion development.

The first limitation of this analysis is that patients might not have reported exposures because of social desirability or recall bias. Rectal and oropharyngeal exams were not conducted, so it is possible that lesions in those anatomic sites went undetected. Most patients with HIV infection were virally suppressed, so our findings may not be applicable to immunocompromised patients, who are at greater risk for disseminated rash (*14*). This study was cross-sectional, and therefore lesions that may have developed later were not captured. Finally, small sample sizes resulted in wide CIs for odds ratios.

Lesion presence, quantity, and onset site may be proxies for identifying the anatomic site of MPXV exposure. However, lesions may appear at anatomic sites where exposures did not occur and may be absent where exposures did occur. Our findings highlight the importance of clinicians conducting a complete physical examination, including a thorough skin and mucosal examination, for patients with suspected mpox. Patients with suspected mpox should be aware that lesions may occur first at mucosal sites, particularly at the sites of exposure. Findings from this study reinforce public health guidance about mpox prevention by avoiding close, skin-to-skin contact with persons who have a rash (*15*).

Appendix Additional information about analysis of monkeypox virus exposures and lesions by anatomic site.
