# Supplementary material for: Analysis of Monkeypox Virus Exposures and Lesions by Anatomic Site
Source: Emerg Infect Dis. 2024 Nov;30(11):2381–4. doi: 10.3201/eid3011.241120 (PMC11521176; doi:10.3201/eid3011.241120)
Supplement: Appendix — Additional information about analysis of monkeypox virus exposures and lesions by anatomic site. [file 24-1120-Techapp-s1.pdf]

Article DOI: https://doi.org/10.3201/eid3011.241120

EID cannot ensure accessibility for supplementary materials supplied by authors. Readers who have difficulty accessing supplementary content should contact the authors for assistance.

Analysis of Monkeypox Virus Exposures and Lesions by Anatomic Site

Appendix

Appendix Table. Univariable logistic regression models assessing the relationship between exposures lesion presence/absence and lesion onset site, among patients who reported an exposure.

				Univariable Models			
N*	Outcome†	Predictor	n‡	**OR**	**(95% CI)**	**p**	**AIC**
216	Penis lesions (n = 95)	Penis exposure	138	4.8	(2.5–9.4)	<0.0001	269.2
216	Anal lesions (n = 59)	Rectum/anus exposure	110	4.8	(2.4–9.9)	<0.0001	230.5
216	Mouth lesions (n = 42)	Mouth exposure	116	1.4	(0.7–2.9)	0.32	213.6
216	Penis lesions first (n = 38)	Penis exposure	138	4.6	(1.9–13.9)	<0.005	193.0
216	Anal lesions first (n = 29)	Rectum/anus exposure	110	2.4	(1.1–5.8)	<0.05	169.9
216	Mouth lesions first (n = 24)	Mouth exposure	116	0.8	(0.4–2.0)	0.7	154.6

AIC, Akaike Information Criterion; OR, Odds Ratio
*The number of observations included in the regression model.
†The number of patients with lesions at this anatomic site.
‡The number of patients exposed at this anatomic site.
